# Socio-demographic factors associated with delayed childbearing in Nigeria

**DOI:** 10.1186/s13104-019-4414-x

**Published:** 2019-07-01

**Authors:** Bola Lukman Solanke, Omowunmi Romoke Salau, Oluwafeyikemi Eunice Popoola, Munirat Olayinka Adebiyi, Olayinka Oluseyi Ajao

**Affiliations:** 1 Department of Demography and Social Statistics, Obafemi Awolowo University, Ile-Ife, Nigeria; 2 Department of Nursing Science, Obafemi Awolowo University, Ile-Ife, Nigeria; 3 Department of Nursing Science, Osun State University, Osogbo, Nigeria; 4 Obafemi Awolowo University Teaching Hospital Complex, Obafemi Awolowo University, Ile-Ife, Nigeria

**Keywords:** Delayed childbearing, Maternal, Pregnancy, Women, Nigeria

## Abstract

**Objective:**

Delayed childbearing is an emerging public health issue in developing countries compared with more developed countries, where it is already a major clinical and public health concern. Previous studies have mostly focused on either the health risks associated with delayed childbearing or the reasons for it with little done around the socio-demographic factors associated with it in developing countries. The objective of the study was to examine associated socio-demographic factors of delayed childbearing in Nigeria.

**Results:**

The study used secondary data pooled from 2003 to 2013 Nigeria Demographic and Health Surveys. The outcome variable was delayed childbearing. The explanatory variables are selected individual socio-demographic characteristics and community characteristics. A weighted sample size of 20,550 women was analysed. Results showed a prevalence of 8.0% delayed childbearing in Nigeria. Socio-demographic factors such as higher maternal education, age at first marriage of 25 years or older, modern contraceptive use, and remarriage status were significantly associated with delayed childbearing. Significant associations were also observed with high community literacy level and high proportion of women who ever used modern contraceptive in the community.

## Introduction

Delayed childbearing refers to first pregnancy or childbirth occurring in women aged 35 years or older [1, 2]. Delayed childbearing has fully emerged as a serious clinical and public health issue in more developed countries. It substantially contributed to the phenomenon of advanced maternal age pregnancy, which is not only widely associated with elevated risk of adverse maternal and child health [3–6], but has also been linked to involuntary childlessness [7, 8]. In Nigeria, the prevalence of delayed childbearing has not received much research or population policy attention [9] due to the persistence of high-risk births among women of reproductive age [10]. Nevertheless, delayed childbearing is emerging in Nigeria due to changing social structures including rise in single parenthood, increased female access to higher education, and increasing professional career enhancement among Nigerian women. Hence, there is need for research to provide more public health education on delayed childbearing before it reaches epidemic proportions in the country.

In Nigeria, studies have rarely investigated delayed childbearing. Though, a Nigerian study examined women with delayed childbearing [11]. Findings from the study indicated that delayed childbearing was not correlated with adverse pregnancy outcomes, which contradicted most other studies [4–6]. However, the study may not have appropriately reflected the Nigerian situation because it was based on a single hospital record and limited only to one out of the 36 states of the country. While most studies have examined different health implications of delayed childbearing [12–14], other studies have focused on the reasons for delayed childbearing [15]. These studies rarely pay attention to the associated socio-demographic factors of delayed childbearing. This has limited understanding of the socio-demographic factors that are needed to be targeted in the provision of counselling and management of women delaying childbearing in a highly parous setting like Nigeria. The objective of the study was therefore to examine the association between socio-demographic factors and delayed childbearing in Nigeria. The Second Demographic Transition Theory [16, 17] underpinned the study. The theory asserts that changing demographic and societal characteristics will result in fertility postponement.

## Main text

### Methods

#### Data source and sample

The study was based on the analysis of secondary data pooled from the 2003–2013 Nigeria Demographic and Health Surveys (NDHS). The pooling generated a large sample size sufficient for drawing valid inference on the socio-demographic factors associated with delayed childbearing. This technique has been adopted in a number of studies that analysed Demographic and Health Survey (DHS) data [18]. Comprehensive details of the DHS design and methodology are widely available [19–21]. This study analysed a weighted sample of 20,550 women aged 35–49 years.

#### Research variables

The outcome variable was delayed childbearing. This was measured by either being currently pregnant with first child at age 35 years or older or having had the first child birth at age 35 years or older in line with description of delayed childbearing [1, 6, 22]. The explanatory variables are sets of individual socio-demographic characteristics selected based on literature providing reasons for delayed childbearing [1, 23]. Community level characteristics such as type of community, community literacy level, community wealth level, proportion ever used modern contraceptive in community, and geographic region were included as explanatory variables based on research evidence that community characteristics have independent effects on reproductive and health behaviour [24, 25]. Three household characteristics, namely household wealth quintile, remarriage and living arrangement were selected as control variables in the study.

#### Data analysis

The study utilised a two-level data analysis procedure. The first level involved the use of cross tabulations of delayed childbearing and the explanatory variables, and with the use of binary logistic regression coefficients to determine whether a positive or negative relationship exist between the research variables. At the second level, the multilevel logistic regression model was applied. The multilevel logistic regression was suitable for the study because the individual women being analysed are nested within the clusters, which were the primary sampling unit in the surveys. Also, this method is suitable when there are multiple levels of influence on the outcome variable [26] as obtained in this study, where the individual characteristics are the lower level of influence and the community characteristics are the higher level of influence on delayed childbearing. The significance of the higher level of influence was assessed using the intra-cluster correlation (ICC), which ranges from zero to one, but may be expressed in percentages. The closer the ICC is to one, the stronger is the higher level influence on delayed childbearing [27]. Three multilevel logistic models were fitted. Model 1 was based on individual characteristics, while community characteristics were included in Model 2. The full model (Model 3) included individual, community, and the control variables. Statistical significance was accepted at p < 0.05. All analyses were performed using Stata version 14 [28].

#### Ethical consideration

The National Health Research Ethic Committee (NHREC/01/2007) approved the NDHSs in Nigeria. The analyses are in anonymised form, and thus not offensive to any individual or community. The datasets are available for use by the general public.

### Results

Result revealed a prevalence of eight percent delayed childbearing among the women. Table 1 presents results of the bivariate analysis. Maternal age was significantly negatively associated with delayed childbearing with higher prevalence of delayed childbearing occurring in the 35–39 reproductive age group (12.0%). The prevalence of delayed childbearing was highest among women who married at age 25 years or older ages (14.5%). Education had mixed relationship with delayed childbearing. Female autonomy on household decision was positively and significantly associated with delayed childbearing with higher prevalence of delayed childbearing among women who had full autonomy on household decisions. As contraceptive use among the women improved from traditional to modern method, the prevalence of delayed childbearing also improves substantially.

**Table 1:** Prevalence of delayed childbearing by socio-demographic characteristics and association by binary logistic coefficients

Characteristic	Prevalence	Coef.	p-value	Characteristic	Prevalence	Coef.	p-value
Reproductive age				Living arrangement			
35–39 years^RC^	12.0	–	–	Living together^RC^	8.5	–	–
40–44 years	7.9	− 0.466	p < 0.01	Living elsewhere	7.5	− 2.515	p < 0.01
45–49 years	3.6	− 1.310	p < 0.01	Household wealth quintile			
Age at first marriage				Poorest^RC^	9.3	–	–
14 years or younger^RC^	7.3	–	–	Poorer	9.8	0.060	0.468
15–19 years	8.0	0.099	0.176	Middle	8.4	− 0.118	0.177
20–24 years	6.7	− 0.079	0.373	Richer	7.0	− 0.310	p < 0.05
25 years or older	14.5	0.776	p < 0.01	Richest	7.2	− 0.278	p < 0.05
Education				Community literacy level			
None^RC^	8.7	–	–	Low^RC^	7.2	–	–
Primary	7.7	− 0.135	0.070	Medium	8.6	− 1.310	p < 0.01
Secondary	7.6	− 0.144	0.094	High	9.5	− 1.989	p < 0.01
Higher	9.7	0.119	p < 0.05	Community wealth level			
Female autonomy				Low^RC^	9.3	–	–
Full autonomy^RC^	9.3	–	–	Middle	8.4	− 0.115	0.105
Joint autonomy	7.0	0.348	0.076	High	7.6	− 0.225	p < 0.05
No autonomy	5.1	0.647	p < 0.01	Proportion ever used modern contraceptive in community			
Current contraceptive use				Low^RC^	7.3	–	–
Not using any method^RC^	1.1	–	–	Medium	8.6	− 0.086	0.232
Using traditional method	2.8	2.401	p < 0.01	High	9.3	− 0.268	p < 0.01
Using modern method	10.1	3.687	p < 0.01	Type of community			
Ideal family size				Urban^RC^	8.9	–	–
1–2 children^RC^	6.0	–	–	Rural	7.4	0.336	p = 0.01
3–4 children	8.2	0.264	p < 0.01	Geographic region			
5 or more children	8.5	0.352	p < 0.05	Northern region^RC^	7.3	–	–
Remarriage				Southern region	9.2	− 2.54	p < 0.01
Married once^RC^	8.2	–	–				
Remarried	9.4	1.157	p < 0.05				

*RC* reference category

p < 0.01 or p < 0.05 (significant)

Results further showed that delayed childbearing was slightly higher among women who had remarried compared to women who married once (9.4% vs. 8.2%). Living arrangement and household wealth quintile were negatively associated with delayed childbearing, with higher prevalence of delayed childbearing among women living together with their partners (8.5% vs. 7.5%). The association between community literacy level and delayed childbearing was negatively significant. On the contrary, the prevalence of delayed childbearing reduced progressively as community wealth level improved from low to high. The association between delayed childbearing and proportion of women ever used modern contraceptive in the community were negatively significant with higher prevalence of delayed childbearing among women currently using modern contraceptive method. The prevalence of delayed childbearing was higher among urban women compared to rural women (8.9% vs. 7.4%). In contrast, geographic zone and delayed childbearing were negatively significantly associated with slightly higher level of delayed childbearing observed in the southern region compared to the northern region.

Table 2 presents the fixed effects on delayed childbearing. In Model 1, with the exclusion of female autonomy and ideal family size, all the included individual characteristics were significantly associated with delayed childbearing. With the inclusion of the community characteristics in Model 2, these variables remained significantly associated with delayed childbearing. Community literacy level and type of community were the only community characteristics that revealed significant association with delayed childbearing in the model. In the full model, women who married at age 25 years or older ages were more than three times more likely to delay childbearing compared to women in the reference category (AOR = 3.571, p < 0.01, CI 2.915–4.375), while women who attained higher education were 48% more likely to delay childbearing compared to uneducated women (AOR = 1.480, p < 0.05, CI 1.130–1.939). Women who had no autonomy on household decisions were 27.8% less likely to delay childbearing compared to women who had full autonomy (AOR = 0.722, p = 0.05, CI 0.576–0.906).

**Table 2:** Fixed effects on the likelihood of delayed childbearing in Nigeria

Characteristic	Model 1 (Wald Chi-sq = 523.6; p < 0.01)			Model 2 (Wald Chi-sq = 522.5; p < 0.01)			Model 3 (Wald Chi-sq = 523.6; p < 0.01)		
	AOR	p-value	95% CI	AOR	p-value	95% CI	AOR	p-value	95% CI
Maternal age (years)									
35–39 years^RC^	–	–	–	–	–	–	–	–	–
40–44 years	0.565	p < 0.01	0.498–0.640	0.565	p < 0.01	0.498–0.640	0.562	p < 0.01	0.496–0.637
45–49 years	0.213	p < 0.01	0.179–0.254	0.215	p < 0.01	0.186–0.257	0.213	p < 0.01	0.178–0.254
Age at first marriage (years)									
14 years or younger	–	–	–	–	–	–	–	–	–
15–19 years	1.195	p < 0.05	1.041–1.372	1.212	p < 0.05	1.055–1.394	1.229	p < 0.05	1.068–1.413
20–24 years	1.264	p < 0.05	1.063–1.505	1.306	p < 0.05	1.095–1.557	1.333	p < 0.05	1.116–1.593
25 years or older	3.316	p < 0.01	2.727–4.031	3.489	p < 0.01	2.855–4.265	3.571	p < 0.01	2.915–4.375
Maternal education									
None^RC^	–	–	–	–	–	–	–	–	–
Primary	1.317	p < 0.01	1.151–1.506	1.078	0.327	0.928–1.252	1.098	0.235	0.941–1.281
Secondary	1.433	p < 0.01	1.208–1.701	0.991	0.925	0.821–1.196	1.053	0.614	0.862–1.286
Higher	3.414	p < 0.01	2.870–4.060	1.333	p < 0.05	1.043–1.702	1.480	p < 0.05	1.130–1.939
Female autonomy									
Full autonomy^RC^	–	–	–	–	–	–	–	–	–
Joint autonomy	0.970	0.708	0.829–1.136	0.078	p < 0.01	0.046–0.132	0.917	0.255	0.791–1.064
No autonomy	0.801	0.082	0.624–1.028	0.013	p < 0.01	0.005–0.035	0.722	p = 0.05	0.576–0.906
Current contraceptive use									
Not using any method^RC^	–	–	–	–	–	–	–	–	–
Using traditional method	1.227	p < 0.05	1.047–1.439	1.329	p = 0.05	1.092–1.617	1.043	0.513	0.919–1.183
Using modern method	1.402	p < 0.05	1.114–1.764	1.737	p < 0.01	1.435–2.102	1.264	p = 0.01	1.108–1.443
Ideal family size									
1–2 children^RC^	–	–	–	–	–	–	–	–	–
3–4 children	1.323	0.193	0.867–2.019	1.385	0.133	0.906–2.117	1.400	0.121	0.914–2.144
5 or more children	1.455	0.063	0.980–2.161	1.468	0.058	0.987–2.184	1.476	0.055	0.991–2.197
Community literacy level									
Low^RC^				–	–	–	–	–	–
Medium				1.133	0.179	0.944–1.360	1.181	0.091	0.973–1.434
High				1.426	p < 0.01	1.210–1.681	1.286	p < 0.05	1.099–1.504
Community wealth level									
Low^RC^				–	–	–	–	–	–
Medium				0.927	0.313	0.801–1.073	0.940	0.417	0.811–1.091
High				1.029	0.776	0.843–1.257	1.068	0.534	0.868–1.313
Proportion ever used modern contraceptive in community									
Low^RC^				–	–	–	–	–	–
Medium				1.006	0.928	0.875–1.158	0.975	0.729	0.846–1.124
High				1.014	0.878	0.849–1.211	1.440	p < 0.01	1.205–1.720
Type of community									
Urban^RC^				–	–	–	–	–	–
Rural				0.822	0.001	0.733–0.922	0.945	0.389	0.830–1.075
Geographic region									
Northern region^RC^				–	–	–	–	–	–
Southern region				0.904	0.237	0.765–1.069	0.916	0.311	0.774–1.085
Household wealth									
Poorest^RC^							–	–	–
Poorer							1.088	0.293	0.929–1.274
Middle							1.041	0.653	0.872–1.243
Richer							0.920	0.442	0.745–1.137
Richest							0.895	0.409	0.688–1.164
Remarriage									
Married once							–	–	–
Remarried							1.222	p < 0.05	1.067–1.400
Living arrangement									
Living together							–	–	–
Living elsewhere							0.886	0.214	0.731–1.072

*RC* reference category

p < 0.01 or p < 0.05 (significant)

Also, women currently using modern contraceptives were 26.4% more likely to delay childbearing compared to women not using any method (AOR = 1.264, p = 0.01, CI 1.108–1.443). Women in communities with high proportion of women who had at least secondary education were 28.6% more likely to delay childbearing compared to women in the reference category (AOR = 1.286, p < 0.05, CI 1.099–1.504). Women in communities with high proportion of women who had ever used modern contraceptive were 44.0% more likely to delay childbearing compared to women in the reference category (AOR = 1.440, p < 0.01, CI 1.205–1.720). Likewise, remarried women were 22.2% more likely to delay childbearing compared to women who married once (AOR = 1.222, p < 0.05, CI 1.067–1.400). As shown in Table 3, the results of the ICC revealed that the community characteristics were responsible for some of the variations in delayed childbearing, though the attributable influence of the community characteristics are not substantial.

**Table 3:** Multilevel logistic regression showing random-effects on delayed childbearing

Parameter	Empty model			Model 1			Model 2			Model 3		
	Est.	S. Err.	95% CI	Est.	S. Err.	95% CI	Est.	S. Err.	95% CI	Est.	S. Err.	95% CI
Variance (community)	0.808	0.103	0.63–1.04	0.769	0.104	0.59–1.01	0.896	0.081	0.75–1.07	0.580	0.037	0.51–0.66
Variance (individual)	0.931	0.070	0.08–1.08	0.575	0.036	0.51–0.65	0.188	0.069	0.05–0.32	0.241	0.022	0.20–0.29
Log likelihood	− 5883.1			− 5281.9			− 5275.8			− 5268.9		
1CC (%)	19.7			14.9			21.4			15.0		
LR test	χ^2^ = 18.45; p < 0.05			χ^2^ = 24.61; p < 0.05			χ^2^ = 24.48; p < 0.05			χ^2^ = 35.07; p < 0.05		

### Discussion

This study is novel because it shifts focus from the more obvious persistent high-risk births which has attracted the attention of previous studies in the country [10] to an emerging social and health issue in the country. The study provided evidence that beyond individual-level characteristics, community-level characteristics such as community literacy level are also associated with delayed childbearing. Findings from the study provided support for the Second Demographic Transition Theory [16, 17] by revealing the significant associations of higher education, female autonomy, modern contraceptive use, and higher age at marriage with delayed childbearing. Though, Nigeria is yet to complete the first demographic transition, the features of the second demographic transition such as single parenthood [29], rise in divorce, and women’s career advancement are increasingly manifesting in the country with likely implications for fertility and childbearing behaviour. Due to increasing gender advocacy in the country, many young Nigerian women are postponing marriage for the purpose of completing higher education programmes or building professional careers. They may therefore, not be willing to commence childbearing until some future date which may put them at high risk of having delayed pregnancy in the absence of knowledge of the risk of fertility postponement. This group of young women need information, education and communication to avoid the risks of delayed childbearing [23] and to ensure that their reproductive choices are informed.

This study revealed delayed childbearing prevalence of less than one-tenth among women in advanced reproductive ages in Nigeria. Though, this prevalence is low, it however, indicates that maternity healthcare delivery system in the country should begin to anticipate a rising likelihood of delayed childbearing with its accompanying adverse effects on maternal and child health [11, 12, 14], and thus initiate steps to curtail further rise of delayed childbearing in the country. This is particularly important because reproductive technology that could be used to manage the health consequences of delayed childbearing are not yet and widely available in Nigeria unlike in Europe and North America [1], and in the few places where the technology is available in the country, it is expensive to access.

Such initiative which may be integrated into existing population and health programmes such as the national family planning programme [9], and should seek to raise awareness of the health risk involved in delayed childbearing. This may be of help to many women who are delaying childbearing not as a result of personal choice [2] but rather as a result of lack of adequate and comprehensive information on its likely health risks. As revealed in this study, users of modern contraceptives had higher likelihood of delayed childbearing. This call for a repositioning of the family planning programme to capture this reality and to provide appropriate education in this regard.

The study concluded that the prevalence of delayed childbearing is currently low in Nigeria. The socio-demographic characteristics significantly associated with delayed childbearing in the country are age at marriage, education, female autonomy, modern contraceptive use, community literacy level, and proportion of women ever used modern contraceptive in community. These characteristics should be targeted in interventions to curtail the rise of delayed childbearing in Nigeria. It is important to reposition family planning programme in the country by expanding existing strategies to include information, education and communication on consequences of delayed childbearing.

## Limitations

In spite of the numerous strengths of the study, a number of drawbacks were identified. First, the dataset analysed do not provide information on the reasons for delayed childbearing among the sampled women. The study is thus not able to exclude women who had delayed childbearing due to fertility challenges. Second, the possibility of recall bias in the responses cannot be overruled because the survey relied on self-reporting. Third, the study was limited to examining association between socio-demographic factors and delayed childbearing due to the use of cross-sectional data which did not allow the study to establish a cause-effect relationship between the variables.

## Data Availability

The dataset supporting the conclusions of this article is available online at https://dhsprogram.com/data/.

## Abbreviations

DHS: Demographic and Health Survey
NDHS: Nigeria Demographic and Health Survey
